# Sex differences in dynamic and static measures of brain integration derived from resting-state functional magnetic resonance imaging

**DOI:** 10.1186/s13293-026-00891-z

**Published:** 2026-04-04

**Authors:** Xiaojing Fang, Olivia Schwemmer, Abigail Hogan, Michael Marxen

**Affiliations:** 1 Department of Psychiatry and Psychotherapy, Technische Universität Dresden, Würzburger Straße 35, 01187 Dresden, Germany; 2 Department of Psychology, Technische Universität Dresden, Würzburger Straße 35, 01187 Dresden, Germany

**Keywords:** Resting-state fMRI, Dynamic functional connectivity, Sliding window analysis, Sex difference, Functional integration, Functional segregation, Human brain connectome

## Abstract

**Background:**

Understanding the impact of biological sex on the functional organization and dynamics of the brain is crucial for elucidating sex-specific differences in cognitive functions and neuropsychiatric disorders. Systems neuroscience often models the brain as a network of interconnected brain regions with functional connectivity (FC), i.e., the correlation between signal time courses, serving as a measure of connection strength. FC matrices, here derived from resting-state functional magnetic resonance imaging (rs-fMRI), define a network graph that can be characterized by its level of module segregation or, inversely, integration. Such parameters can be generated for the full length of the acquired data (static) or for short periods implying dynamically changing brain states. We recently made the interesting observation in a separate study (*N* = 63) that measures of brain integration and segregation based on dynamic functional connectivity (dFC) data differed between sexes, while graph-based measures based on static FC (sFC) did not, which we investigated in more detail in this study.

**Methods:**

We preregistered a replication of our analysis from the small sample in *N* = 501 subjects of the Human Connectome Project dataset. We performed cross-sectional comparisons between sexes of the static rs-fMRI graph parameters modularity and global efficiency, as well as the dFC parameters state prevalence, mean dwell time, mean inter-state transition time, and variability derived from a two-state model. Additionally, we explored whether sex differences in 66 cognitive and behavioral parameters are mediated by the FC integration measure with the strongest sex effect.

**Results:**

All static and dynamic measures of integration/segregation showed higher levels of functional integration in males, with effect sizes up to 0.60 for the dFC parameter prevalence. For three of the 66 explored cognitive and behavioral parameters, we observed that the prevalence of the integrated state mediated the sex difference: dexterity, agreeableness, and self-reported aggression.

**Conclusion:**

We found consistent evidence across two datasets that rs-fMRI-based measures of brain integration are increased in males. An exploratory analysis, which requires replication, suggests that such differences mediate personality differences. This study highlights that biological sex differences in brain functional organization may contribute to sex-typical behaviors.

**Supplementary Information:**

The online version contains supplementary material available at 10.1186/s13293-026-00891-z.

## Abstract (plain language summary)

Functional connectivity measures the similarity of activity between two brain regions over time. It is thought to be a measure of coupling strength between these regions and is used to define functional networks or modules of multiple brain regions. This aspect of brain architecture is analogous to public transportation networks between the cities of a country. This architecture can be presumed to be stable over minutes (static) or change every couple of seconds (dynamic). Both approaches can be used to define how integrated the modules of the brain are, which is a property of the underlying architecture. While it is known that this architecture differs between women and men, the ways in which it is different, and the consequences of such differences are unclear.

In this study, we show that men have higher levels of modular integration than women in both static and dynamic measures of this property. We also demonstrate that such differences may lead to known variations in behaviors, such as agreeableness and self-reported aggression. This is an important step forward in our endeavor to understand whether and how differences in brain structure and function between the sexes may lead to or contribute to known differences in behavior.

**Supplementary Information:**

The online version contains supplementary material available at 10.1186/s13293-026-00891-z.

## Highlights

- Males show higher brain integration than females in static and dynamic parameters.
- Sex differences were stronger in dynamic than static parameters.
- Brain integration mediated sex effects in dexterity, agreeableness and aggression.
- Replication in N = 501 confirmed preregistered findings in N = 63.
- The results help to understand the neural basis of behavioral sex dimorphism.

**Supplementary Information:**

The online version contains supplementary material available at 10.1186/s13293-026-00891-z.

## Introduction

Studying differences between biological sexes with respect to intrinsic functional brain organization, in addition to other biological, environmental and sociocultural variables, enhances our comprehension of individual differences in subsequent behavior and cognition [1, 2] in healthy individuals and neuropsychiatric conditions [3]. This could inform tailored interventions and personalized treatment strategies in various psychiatric and neurological disorders with sex disparities [4, 5]. Functional connectivity (FC) is a measure of functional brain organization that captures the synchrony of neural activity time series across distinct brain regions. Traditionally, FC is calculated across the full length of an fMRI resting-state scan (usually 5–10 min.). Going beyond this static functional connectivity (sFC) approach, investigating FC in a dynamic fashion (i.e., dynamic functional connectivity; dFC) [6] has become more and more prevalent in recent years as this method is capable of accounting for the temporal variations in synchrony across the resting-state time course [7, 8].

The general existence of sex differences in sFC has been established in the field. Early functional studies reported that resting-state FC is more efficient in the right hemisphere of males and in the left hemisphere of females [9, 10], suggesting that sex differences in cognition may, in part, be related to divergent neural patterns in the brain [11]. Moreover, stronger FC within the default mode network and reduced FC in sensorimotor cortices during rest have been reported in females [12, 13]. In contrast, reports on sex differences in dFC measures are still rare, possibly due to the novelty and greater complexity of the approach. A few studies focused on classification accuracy but did not report specific FC differences [14, 15]. Two reports investigated sex differences in dFC parameters using 4-state models [3, 16], one of which suggested a possible link between differential neurocognitive performance in males and females and brain functional dynamics [3]. However, to our knowledge, no study has provided evidence that sex differences in behavior are actually mediated by sFC or dFC parameters.

For our dFC analysis, we have opted for a two-state model. This model offers good reliability of states and the associated prevalence parameter, which degrades with a higher number of states [17]. The two-state model also offers the conceptual advantage that one of the states corresponds to a more integrated graph network, the other to a more segregated one [17]. The idea that FC patterns may be described by the mean path length between pairs of brain regions (integration/global efficiency) or the fraction of within-module versus between module connections (segregation/modularity) goes back to Friston [18] and was previously utilized in the context of dFC, for example, by Shine et al. [19]. It offers an intuitive description of functional architecture and can also be quantified by the static FC graph-theoretical parameters global efficiency (increasing with functional integration and decreasing with functional segregation) and modularity (increasing with functional segregation and decreasing with functional integration), respectively (see parameter correlations in Table S1).

Based on findings in a small in-house study (OWN; *N* = 63) with limited power [20], we preregistered the following hypotheses to be tested in a much larger sample, i.e., 501 subjects from the publicly available human connectome project (HCP) [21, 22]: higher prevalence of the segregated state (*Prev*_*S*_) (H1) and longer mean dwell time of the segregated state (*MDT*_*S*_) (H2), and lower variability of the integrated state (*Var*_*I*_) (H3) in females than in males. With respect to sFC parameters, we hypothesized lower global efficiency (H4) and greater modularity (H5) in females than in males. Confirmation of H1, H2, H4, and H5 would support the notion that men show higher levels of brain integration than women. Finally, with respect to relative effect sizes, we hypothesized that effect sizes for the sFC parameters would be smaller than for the dFC parameter *Prev*_*S*_ (H6). Additionally, we conducted an exploratory mediation analysis of 66 cognitive and behavioral measures to test whether sex disparities are mediated by brain integration.

## Methods

**Fig. 1:**
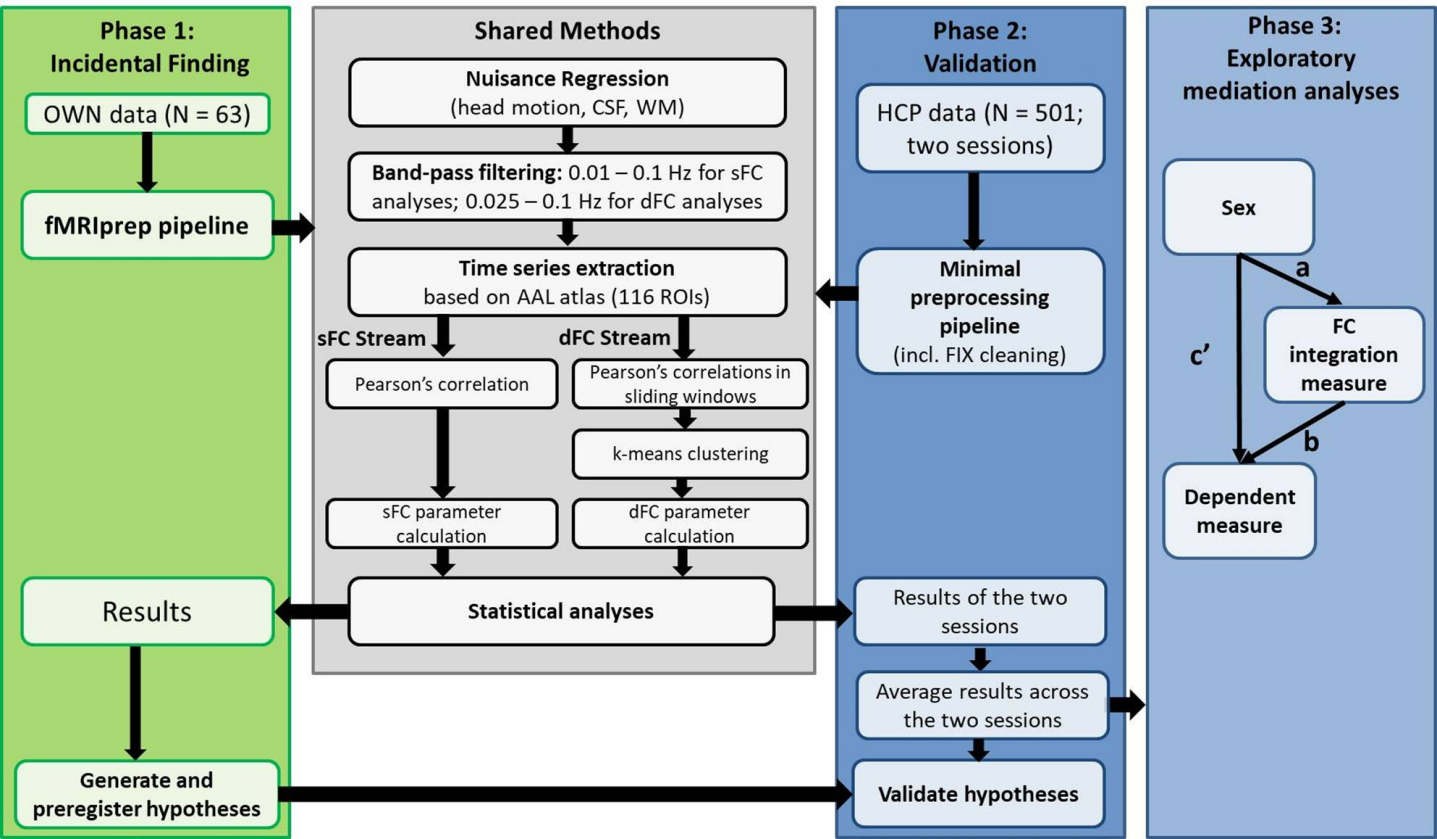
Data processing flow chart. Initially, we observed a sex effect on dFC parameters in our OWN data that inspired us to preregister hypotheses (Phase 1) and validate these in a larger data set (Phase 2). We used nearly identical processing methods. In Phase 3, we investigated whether sex differences in a number of cognitive/behavioral parameters are mediated by the brain FC integration measure that showed the largest sex effect. (OWN – label for the data acquired within our center; HCP – Human Connectome Project; sFC – static functional connectivity; dFC – dynamic functional connectivity; CSF – cerebral spinal fluid; WM – white matter; a – effect of sex on the integration parameter; b – effect of integration parameter on the cognitive/behavioral dependent parameter; c’ – direct effect of sex on the dependent parameter; a*b – indirect mediation effect)

An overview of the analyses conducted is provided in the workflow diagram in Fig. 1. Details can be found in the sections below and the Supplemental Information.

### Participants and data acquisition

We report data from two studies (see Table 1) here: the initial findings stem from our OWN data (*N* = 63) [23], whereas the confirmatory and preregistered analyses [22] were performed on HCP data (*N* = 501) [21, 22].

#### OWN data

Within the Collaborative Research Center (CRC) 940 on *Volition and Cognitive Control* funded by the Deutsche Forschungsgemeinschaft (DFG), 80 subjects of subproject C1 on *Volitional Dysfunctions in Self-Control Failures and Addictive Behaviors* [24] agreed to participate in an additional session of MRI scans. The MRI acquisition parameters were the same as those reported in a previous study [17] with a duration of the rs-fMRI scan of 16 min 27 s (see Supplementary Information).

Regarding participant exclusions, eleven participants were excluded because more than 7.5% of frames had framewise displacements (FD) of 0.5 mm or more, five were missing behavioral data needed for the original investigation and one participant was excluded because of problems with normalization to MNI space. Among the resulting *N* = 63 participants, 49% met the criteria of Diagnostic and Statistical Manual of Mental Disorders (Fifth Edition) [25] for a mostly mild, addictive disorder, and 51% were healthy controls. All participants received financial compensation after MRI data collection. The study was approved by the Ethics Committee of the Technische Universität Dresden (EK 4012016) and all participants signed informed consent forms after receiving a detailed description of the experiment. This sample is also described in the preregistration of the current study [22] in the document “Previous_Results.docx”.

#### HCP data

As preregistered [22], HCP data consisted of 501 subjects with two scanning sessions each from the HCP S1200 release [26], which we used previously in another study [17]. The duration of the rs-fMRI scan was 14 min 24 s in each session (see Supplementary Information for acquisition parameters).

### Preprocessing

For OWN data, the preprocessing pipeline used fMRIPrep 1.2.5 (zenodo.org/record/4252786#.X7TzMGhKhPZ) based on Nipype 1.1.6 (zenodo.org/record/4035081#.X7Ty32hKhPY) [27], as in the previous study [17]. HCP data was used with the provided preprocessing. See Sect.  1.2 in the Supplementary Information for more details.

### FC measures

The atlas used for FC analyses is described in Sect.  1.3 in the Supplementary Information. Static FCs were computed as the Pearson correlation z-values based on the ROI-wise time series. To quantify static functional segregation and integration, graph theory-based parameters of modularity and global efficiency (see Sect.  1.4 in the Supplementary Information) were computed in the brain connectivity toolbox (BCT) [28, 29].

dFC was calculated as Pearson correlation *z*-values for each window frame (i.e., brain state instance) via SWA in DynamicBC toolbox [30] with a step size of 1 repetition time (TR). For SWA, a window size of 40 TRs = 39.48 s in the OWN data and 55 TRs = 39.60 s in the HCP data were chosen, taking the difference in TR between studies (987 ms versus 720 ms) into account and matching the time-window lengths in seconds as closely as possible (see also [17]). For clustering, we used k-means (k = 2) with cosine distance to classify brain state instances as functional integration or segregation (i.e., states I or S) for all dFC matrices. For the OWN data, the extraction steps of the brain states are described in our previous work [23], which used the same dataset. For the HCP data, the extraction was the same as in our previous study [17]. We employed mean dwell time (*MDT*), prevalence (*Prev*), inter-transition interval (*ITI*) and state variability (*Var*) (see Sect.  1.5 in the Supplementary Information) based on state I and state S [31] to characterize features of dynamic brain states for each subject [17, 22].

Following our previous methodological study [17], we used a two-state model (i.e., k = 2) in this work. Briefly, silhouette statistics indicated that k = 2 was optimal in 23 subjects with test-retest rs-fMRI data collected with the same parameters as the OWN data [17]. In addition, the selection of k = 2 is advantageous to accurately identify dynamic states, maximize the statistical stability of parameter estimates and maintain clear neurobiological interpretability based on functional segregation and integration [17].

### Statistical analyses

Since some of the dynamic parameters were right-skewed or otherwise not normally distributed, we conducted both independent-samples t-tests and Mann-Whitney U tests on all of the variables. We used different statistical strategies for the parameters in the different datasets. Specifically, because of no directional expectation, the null hypotheses for OWN data tested whether the parameters grouped by sex were significantly (*p* < 0.05) different from zero. Hence, we employed two-sided tests for this dataset [32]. Since the results in the OWN data supported clear directional expectations for the preregistered hypotheses in the HCP data, we used one-sided tests for the parameters modularity, global efficiency, *Prev*_*S*_, *MDT*_*S*_ and *Var*_*I*_. Note that our hypotheses about modularity and global efficiency were not derived from statistically significant findings in the OWN data but based on our hypothesized notion that brain integration measures would be higher in males than in females with too small effect sizes for sFC parameters to reach significance in only 63 subjects (see H6). For all other parameters, two-sided tests were employed [32]. None of the above tests were corrected for multiple comparisons because we regarded them as tests of independent hypotheses.

### Mediation analyses

In an exploratory fashion, we investigated whether sex effects were mediated by brain integration for all 58 behavioral/cognitive parameters provided by the National Institutes of Health Toolbox (http://www.healthmeasures.net/explore-measurement-systems/nih-toolbox) (see Table S2), as used in previous studies [33, 34]. For more details, please refer to the file wiki.humanconnectome.org/docs/assets/HCP_S1200_DataDictionary_Aug_22_2023.csv. Additionally, we examined the effects on eight alcohol consumption parameters (see Table S3), which we selected on the basis of our own research interest in alcohol use disorder. To measure brain integration, we employed *Prev*_*I*_ in line with our hypothesis H6 that dFC parameters would show a stronger sex effect than sFC parameters and the previously reported observation that *Prev* showed the highest test-retest reliability among dFC parameters [17]. This approach aimed to maximize sensitivity when studying whether functional brain integration mediates sex differences in cognitive/behavioral parameters.

Before the mediation analyses, we calculated Pearson correlations of all the dependent NIH and alcohol measures with sex and our measures of brain integration. A two-sample t-test for sex differences was also performed, producing the same *p*-value as the correlation. We then employed structural equation modeling for mediation analysis with 5000 random bootstrapping samples using the *bootstrapLavaan* function from the R software package *lavaan* [35] if the dependent variable was significantly correlated with both sex and the selected integration parameter (*p* < 0.05 after Bonferroni correction for 66 parameters). The dependent measures and the integration measure were standardized (variance = 1). Sex was coded as 1 for males and 2 for females. We computed the direct effect of sex on the dependent measure (i.e., path c’), the influence of sex on the integration measure (i.e., path a) and the influence of the integration measure on the dependent measure (i.e., path b). We reported whether the mediation effect (i.e., a*b) was significantly different from zero on the basis of the 95% confidence interval.

This analysis was exploratory in the sense that we did not have specific hypotheses on which parameters would show sex or mediation effects. When investigating the sex and brain integration effects on the dependent variables, we opted for a conservative Bonferroni correction as larger effects should improve the sensitivity of the mediation analysis. However, we did not correct for the number of performed mediation analyses to avoid false negative findings. Therefore, validation of these observations in other samples will be necessary.

**Table 1:** Demographic information

Dataset	N (female)	Age [years (SD)]	Difference in sex within datasets		Difference in sex between datasets	
			Chi-Square	*p*-value	Pearson Chi-Square	*p-*value
OWN	63 (29)	25.98 (1.61)	0.397	0.529	1.056	0.304
HCP	501 (265)	28.85 (3.63)	1.679	0.195		

SD: standard deviation

**Table 2:** T-tests, Mann-Whitney U-tests, and effect sizes with confidence intervals (CI) comparing individual head motions (median frame-wise displacement [FD]) between males and females in the two datasets

Parameters	Sex	N	Mean FD (SD)	T-test (males – females)		Cohen’s d CI	Median FD	U-test
				*t-*value (df)	*p-*value			*p*-value
**OWN data**	**Male**	34	0.146 (0.041)	0.487(61)	0.628	0.123 [-0.373, 0.619]	0.136	0.730
	**Female**	29	0.141 (0.041)				0.140	
**HCP data**	**Male**	236	0.149 (0.054)	0.033(499)	0.974	0.003 [-0.172, 0.178]	0.137	0.672
	**Female**	265	0.149 (0.046)				0.139	

SD: standard deviation; df: degrees of freedom

## Results

As there was no significant difference in sex distribution within or between the two groups (Table 1), we considered the sex distribution in both datasets to be balanced. Additionally, we extracted individual median FD values and compared the differences between the two groups. The results revealed no significant differences in head motion between the sex groups for the two datasets (Table 2), suggesting that head motion was not a confounder of the results of this study. The centroids (averaged median matrices of individual brain states within groups) for each state and dataset are shown in Figure S1 for OWN data and can be found in our previous publication for HCP data [17].

### Static parameters

#### OWN data

**Fig. 2:**
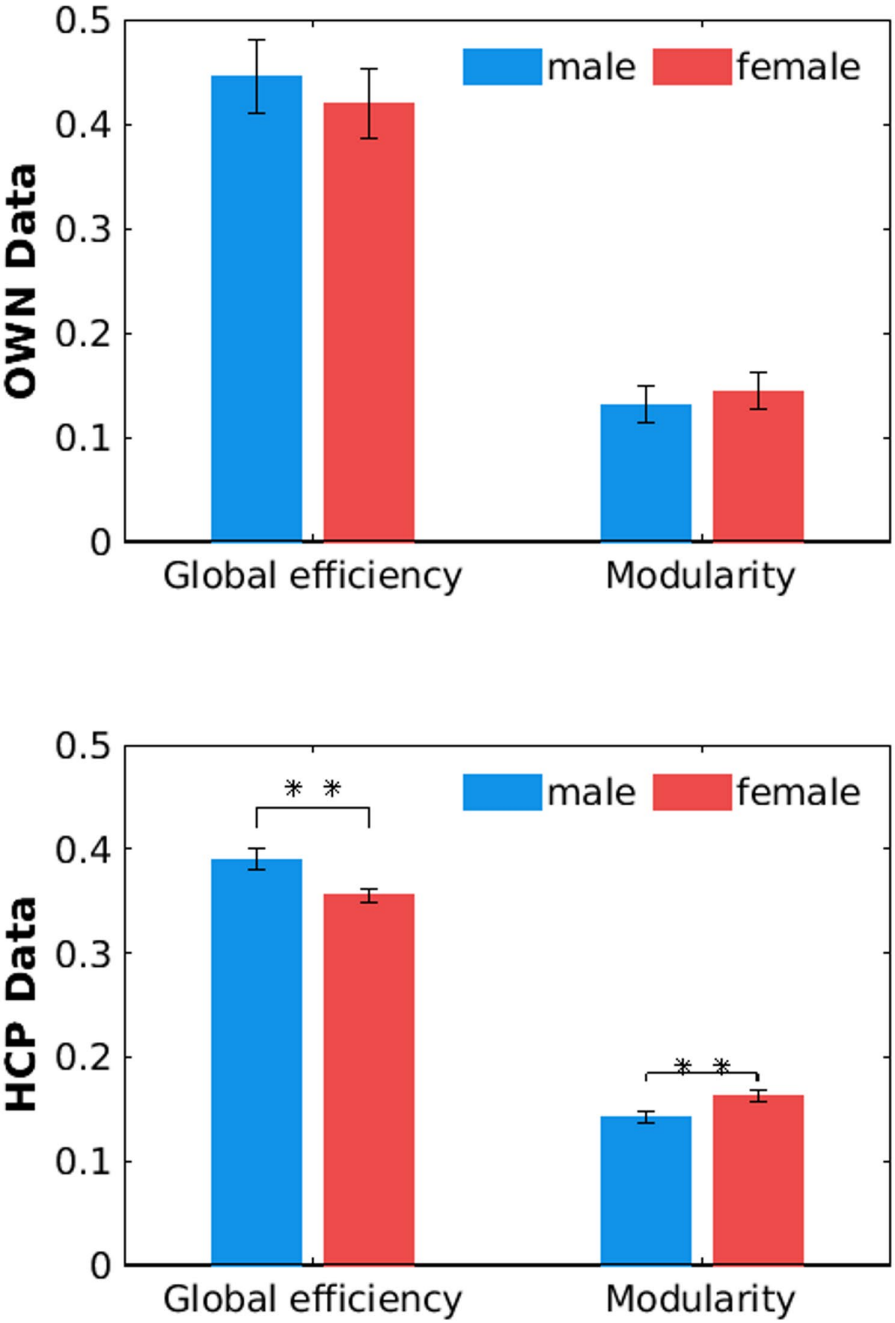
Mean global efficiency and modularity grouped by sex for OWN and HCP data. Error bars: 95% confidence intervals; *: *p* < 0.05, **: *p* < 0.005

There were no significant effects of sex on global efficiency (i.e., a measure of integration) or modularity (i.e., a measure of segregation) in the OWN data (Table 3; upper part in Fig. 2).

#### HCP data

The results revealed significant effects of sex on both global efficiency and modularity (Table 3; lower part in Fig. 2), showing significantly higher global efficiency and lower modularity in males than females, thus confirming hypotheses H4 and H5.

**Table 3:** Results of two-sample t-tests and Mann-Whitney U-tests comparing global efficiency and modularity, two static FC measures of brain integration and segregation, respectively, between females and males for the two datasets

Parameters	sex	*N*	Mean (SD)	t-test (males – females)		Cohen’s d [CI]	Median	U-test
				*t*-value (df)	*p*-value			*p*-value
**OWN data**								
Global efficiency	male	34	0.45 (0.10)	1.079 (61)	0.285	0.273 [-0.226, 0.769]	0.430	0.282
	female	29	0.42 (0.09)				0.403	
Modularity	male	34	0.13 (0.05)	-1.032 (61)	0.306	-0.261 [-0.757, 0.238]	0.126	0.301
	female	29	0.14 (0.05)				0.144	
**HCP data**								
Global efficiency	male	236	0.39 (0.08)	5.632 (444)	0.000**	0.511 [0.333, 0.689]	0.379	0.000**
	female	265	0.36 (0.06)				0.352	
Modularity	male	236	0.14 (0.04)	-5.504 (499)	0.000**	-0.493 [-0.670, -0.314]	0.145	0.000**
	female	265	0.16 (0.04)				0.159	

*: *p* < 0.05; **: *p* < 0.005. SD: standard deviation; *df*: degrees of freedom; underlined parameters in the HCP data: one-sided t-test for hypothesis testing; two-sided t-tests otherwise; *p*-values are uncorrected

### Dynamic parameters

#### OWN data

**Fig. 3:**
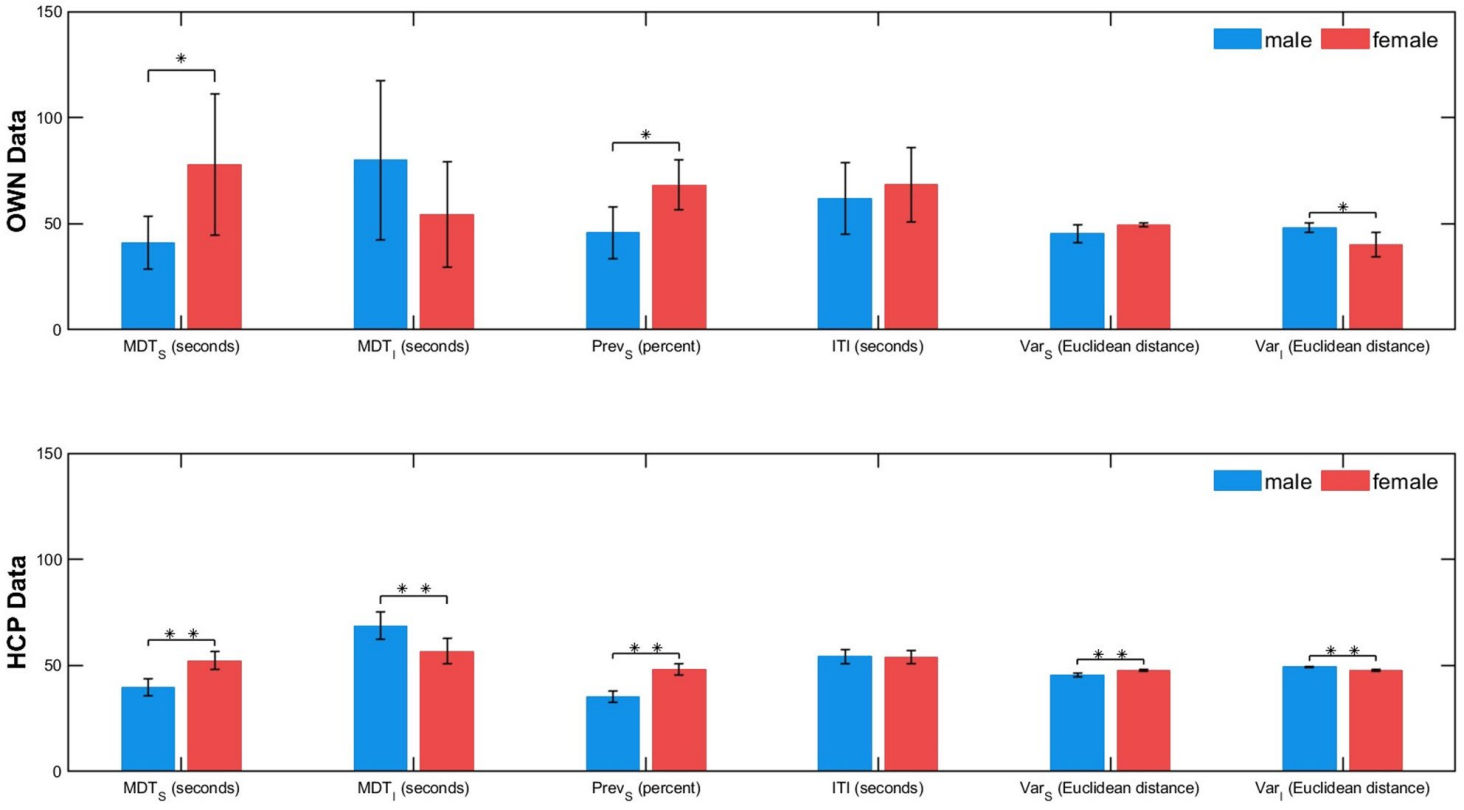
Dynamic FC parameters grouped by sex for the two datasets. Error bars: 95% confidence intervals. The unit of *MDT*_*s*_ and *ITI* is seconds and of *Prev*_*S*_ is percentage; *: *p* < 0.05; **: *p* < 0.005

The results are shown in Table 4 and in the upper part of Fig. 3. *MDT*_*S*_ was significantly greater in females than in males across all tests. *Prev*_*S*_ was significantly greater in females than in males. Notably, as *Prev*_*I*_ = 1- *Prev*_*S*_, this indicates that *Prev*_*I*_ was lower in females. The effect sizes for these parameters are given in Table 4. *Var*_*I*_ was significantly greater in males (*p* < 0.05) (Table 4). There were no significant differences in the other parameters. These results (of two-sided tests) have already been published in our preregistration [22] and are the basis for our hypotheses to be tested in the HCP data.

#### HCP data

We observed significant differences between sexes in *MDT*_*S*_, *MDT*_*I*_, *Prev*_*S*_, *Var*_*S*_ and *Var*_*I*_. Notably, *MDT*_*S*_ and *Prev*_*S*_ were significantly higher and *Var*_*I*_ was significantly lower in females than males, confirming hypotheses H1-3. Additionally, *MDT*_*I*_ was significantly higher and *Var*_*S*_ significantly lower in males than females solely in the HCP dataset. Moreover, similar to the OWN data, there were no significant differences observed for *ITI*. In addition, the *t*-tests with significant results revealed small to medium effect sizes (Table 4). Importantly, the effect size for *Prev*_*S*_ (and thus, by definition for *Prev*_*I*_) was the largest of all observed effects with Cohen’s |d| = 0.6, which was ~ 20% above the effect size for the sFC parameters. This effect size for *Prev* was, however, not significantly different from those for the sFC parameters, as evidenced by the overlapping confidence intervals. Thus, hypothesis H6 could not be confirmed with the available statistical power.

**Table 4:** Results of two-sample t-tests and Mann-Whitney U-tests to compare differences between females and males in dynamic FC parameters from the two datasets

Parameters	sex	*N*	Mean (SD)	*t*-test		Cohen’s d [CI]	Median	U-test
				-value (df)	*p*-value			*p*-value
**OWN data**								
*MDT*_*S*_	male	32	41.09 (34.06)	-2.149 (28.235)	0.040*	-0.658	32.853	0.027*
	female	23	77.92 (76.94)			[-1.205, -0.105]	49.914	
*MDT*_*I*_	male	30	80.06 (100.40)	1.135 (54)	0.261	0.304	50.516	0.286
	female	23	54.37 (61.02)			[-0.226, 0.831]	31.584	
*Prev*_*S*_	male	34	45.76 (35.46)	-2.654 (61)	0.010*	-0.671	42.04	0.007*
	female	29	68.27 (31.16)			[-1.178, -0.159]	75.234	
*ITI*	male	30	61.83 (45.33)	-0.546 (51)	0.588	-0.151	53.621	0.430
	female	23	68.38 (40.52)			[-0.694, 0.394]	55.713	
*Var*_*S*_	male	33	45.44 (11.96)	-1.929 (34.912)	0.062	-0.462	49.62	0.386
	female	29	49.55 (2.40)			[-0.966, 0.045]	49.823	
*Var*_*I*_	male	33	48.14 (5.79)	2.749 (32.994)	0.010*	0.768	48.822	0.019*
	female	27	40.10 (14.26)			[0.237, 1.292]	47.381	
**HCP data**								
*MDT*_*S*_	male	236	39.78 (30.48 )	-4.323 (497.995)	0.000**	-0.385	33.151	0.000**
	female	264	52.24 (34.00)			[-0.562, -0.207]	42.390	
*MDT*_*I*_	male	232	68.76 (49.92)	2.656 (495)	0.008*	0.239	57.440	0.000**
	female	265	56.80 (50.18)			[0.062, 0.416]	43.864	
*Prev*_*S*_	male	236	35.32 (20.91)	-6.721 (499)	0.000**	-0.602	32.853	0.000**
	female	265	48.02 (21.28)			[-0.781, -0.422]	46.466	
*ITI*	male	232	54.22 (24.68)	0.136 (494)	0.892	0.012	47.60	0.717
	female	264	53.91 (24.67)			[-0.164, 0.189]	48.89	
*Var*_*S*_	male	236	45.47 (6.56)	-4.886 (307.272)	0.000**	-0.456	47.578	0.000**
	female	265	47.71 (2.74)			[-0.633, -0.278]	48.315	
*Var*_*I*_	male	236	49.33 (2.21)	6.345 (428.436)	0.000**	0.551	49.390	0.000**
	female	265	47.56 (3.87)			[0.372, 0.730]	48.102	

Effect size Cohen’s d with 95% confidence intervals. The unit of *MDTs* and *ITI* is seconds and of *Prev*_*S*_ is percentage; *: *p* < 0.05; **: *p* < 0.005; SD: standard deviation; *df*: degrees of freedom; CI: 95% confidence interval; underlined parameters in the HCP data: one-sided t-test for hypothesis testing; two-sided t-tests otherwise; *p*-values are uncorrected

### Mediation analyses

After an initial screening based on two-sample t-tests corrected for multiple comparisons, we found that 30 of the 58 behavioral and cognitive parameters and seven of the eight alcohol measures showed sex differences (Tables S2 and S3). Six of these 37 measures all from the former group, showed a bivariate correlation with *Prev*_*I*_ (Table S2): Penn matrix test—number of correct responses (PMAT24_A_CR), short Penn continuous performance test—specificity (SCPT_SPEC), nine-hole pegboard test (Dexterity_Unadj), five-factor model factor summary scores—agreeableness (NEOFAC_A), five-factor model factor summary scores—conscientiousness (NEOFAC_C), and negative affect— sadness, fear, and anger (AngAggr_Unadj). All of them were also correlated with modularity and global efficiency. These six measures were subjected to mediation analyses to test whether the sex effect on *Prev*_*I*_ could explain the sex differences (Table S4).

**Fig. 4:**
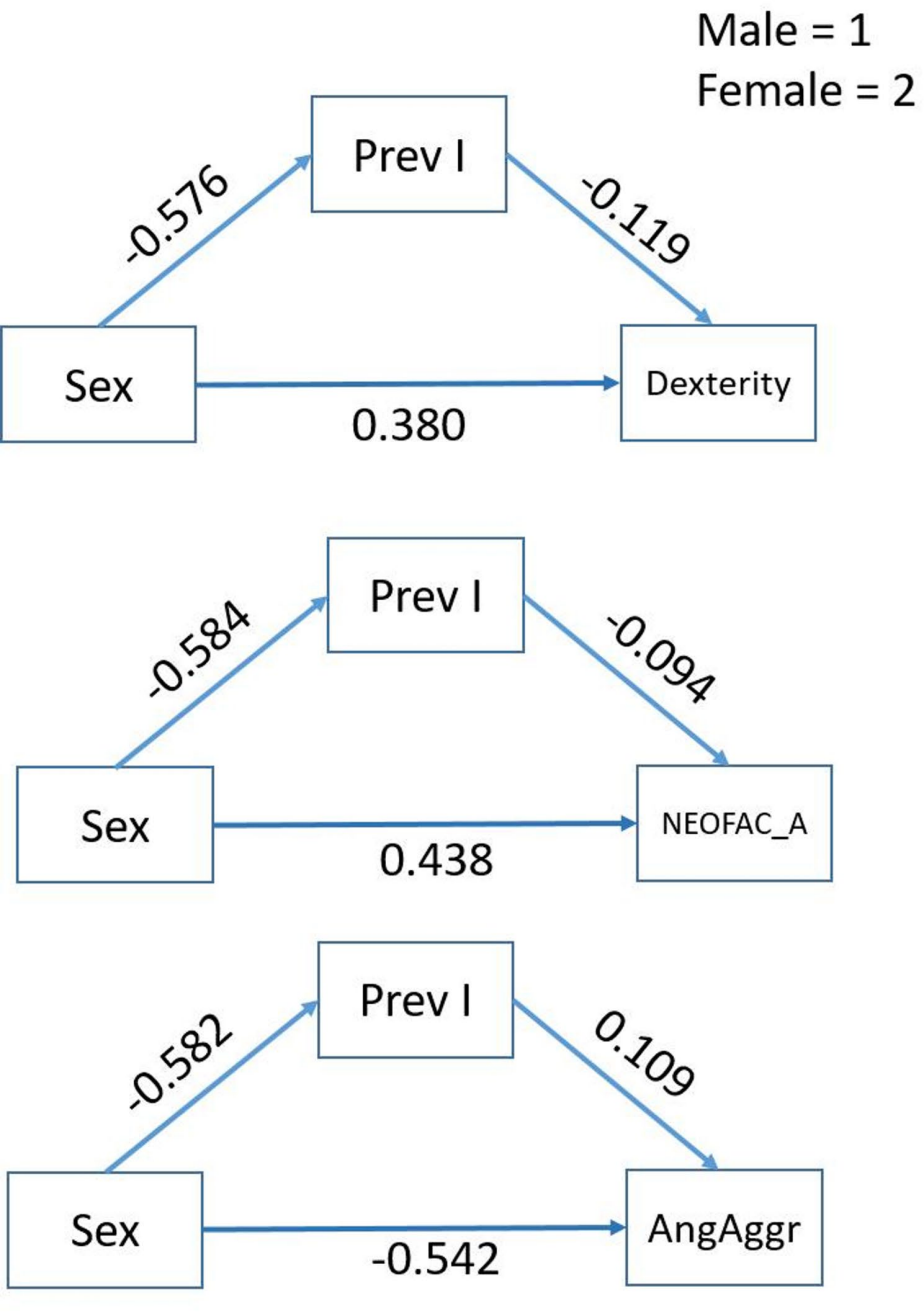
Sex differences identified in behavioral HCP parameters with significant mediation effects via *Prev*_*I*_. All paths in the diagrams are significant (*p*_uncorrected_ < 0.05). Positive numbers for the sex effects imply higher values of the dependent variable for females

The exploratory mediation analyses showed significant [i.e., bootstrapping confidence interval excluded zero] partial indirect/mediation effects of *Prev*_*I*_ in three cases for the effect of sex on dexterity (Dexterity_Unadj), on the agreeableness subscale of the five factor model of human personality NEO-FFI (NEOFAC_A) [36] and on self-reported aggression (AngAggr) (Fig. 4 and Table S4). While the direct sex effects were intermediate, the mediation effects were small.

## Discussion and conclusion

This study investigated biological sex differences in global FC during rest with a focus on markers of functional brain integration and segregation [19], inspired by findings in the OWN data set consisting of 63 subjects [20]. The preregistered hypotheses H1-5 were confirmed in the larger HCP data set with 501 subjects indicating that males tend to show higher measures of brain integration, whereas females tend to show higher measures of segregation. This pattern was observed across multiple static and dynamic FC parameters associated with the concepts of brain integration and segregation. With respect to dFC, our hypotheses H1 to H3 were supported by the findings that females spent more time in segregated brain states (*Prev*_*S [females]*_ = 48% versus *Prev*_*S [males]*_ = 35% for HCP data) (H1) and stayed longer in a segregated brain state before transitioning to an integrated state (*MDT*_*S [females]*_ = 38 s versus *MDT*_*S [males]*_ = 29 s for HCP data) (H2) and shorter in an integrated state (*MDT*_*I [females]*_ = 41 s versus *MDT*_*I [males]*_ = 50 s for HCP data), which was not detected in OWN data, likely due to a lack of statistical power. This reciprocal pattern is consistent with the lack of a sex difference in the time between state switches (*ITI)*, which approximates the mean of the *MDT* times. The results suggest that sex affects the occupancy and duration of brain states rather than the rate of state changes. For the static parameters, we observed higher global efficiency in males, confirming hypothesis H4, and higher modularity in females, confirming hypothesis H5.

While we observed that the dFC parameter *Prev* showed an approximately 20% larger effect size than the static measures modularity or global efficiency, this difference did not reach significance. Thus, hypothesis H6 could not yet be confirmed. A substantially larger sample would be required to detect such an effect size difference (*N* ~ 2500, presuming an effect size of d = 0.1).

Notably, while the dFC parameters *Prev*,* MDT*, and *ITI* are not independent (see Table S1), they reveal different interpretable features of brain state dynamics that cannot be obtained from sFC markers. Consequently, the analysis of dFC measures leads to more insights into the underlying mechanisms of FC group differences. Overall, our findings provide specific and, in part, reproducible evidence for sex differences in resting-state FC, thereby advancing our understanding of sex differences in human brain function. Especially, the reproduction of our findings in the dFC parameters after preregistration (H1-3) in a large cohort and the confirmation of a similar, potentially slightly smaller effect in sFC measures (H4-5) make our findings valuable to the research community in light of justified criticism regarding the limited reproducibility of brain-wide association studies [37]. Related to this issue is also the test-retest reliability of the underlying measures. For our measures, we estimated the intra-class correlation coefficient (ICC) to be approximately 0.5 for *Prev* [17], modularity and global efficiency (see Figure S2) for the HCP sample when using only one acquisition session. Given that the actual values in our study were averaged across the two sessions, these ICC-values are conservative estimates (presuming that the averaging reduces the intra-subject variance by a factor of 2, the ICC-value would rise to 0.67). For neuroimaging data, these values can be considered ‘good’ [38, 39] in terms of our ability to identify brain-sex associations. Yet, the lower reliability of *MDT* times of ICC ≈ 0.2 [17] could be a reason that the true effect sizes for these measures of brain integration would be lower than for the above measures. While we could not confirm this in our samples due to a lack of power, the observed lower effect estimates for *MDT*s are compatible with this hypothesis.

Additionally, we observed sex differences in state variability, i.e. the extent of the within-subject state cloud. *Var*_*S*_ was higher in females, while *Var*_*I*_ was higher in males. This is consistent with the observed correlations between these variables and *Prev* (and *MDT)* of the respective states (Table S1), a correlation that was not induced by the sex effect as it also existed when controlling for sex (Table S5). This association may be explained by presuming that the state distribution across both states of a subject is a single cloud (rather than two disjointed clouds for each state) and that the distributions between sexes (or individuals) are, to first order, merely shifted. Higher values of *Prev*_*S*_ arise when this distribution is shifted toward the centroid of state S. As the boundary between the overall state centroids, however, is fixed, the extent of the segregated state cloud is increased (i.e. *Var*_*S*_). We included *Var* here because it provides a more complete picture of the dFC state distribution beyond the aspect of brain integration and because *Var*_*I*_ showed the largest sex effect of all parameters in the OWN data. In the much larger HCP sample, however, *Prev* showed the largest effect. In summary, considering the coupling with *Prev* suggested above, *Var* does not provide substantial additional insight into the nature of the sex difference in functional brain organization.

Previous ICA-based multi-state studies have reported sex differences in dynamic dwell time, although specific patterns vary across datasets [3, 12, 30]. While some findings broadly overlap with ours at a conceptual level, such as greater network segregation in females and greater integration in males, others, including faster state switching in males, were not observed here [3]. Given the substantial methodological differences in parcellation strategies and the number of assumed states, an in-depth comparison of results is, however, not possible. We chose an atlas-based two-state model prioritizing reproducibility and interpretation in terms of whole-brain integration and segregation.

After identifying sex differences in functional brain organization, it is important to examine whether these differences actually mediate behavioral sex disparities. To this end, we explored this possibility based on a set of 66 cognitive and behavioral measures, without specific hypotheses. First, we identified six variables that exhibited both a sex difference and a correlation with *Prev*_*I*_ (Tables S2 and S3). All six variables were also associated with modularity and global efficiency, in line with the conceptual notion that these measures depend on the level of brain integration. Eventually, we found that the effects of sex on dexterity, NEO-FFI and self-reported aggression were mediated by *Prev*_*I*_ (i.e., bootstrapping CI of the mediation effect excluded 0). However, the mediation effects were small. This is not surprising given that the direct effects of sex on the variables of interest (Fig. 4), as well as the reliability of *Prev*_*I*_ [17], were at best intermediate, and may not survive multiple comparison correction. Thus, these findings need validation in other samples.

A complete understanding of sex differences in functional brain organization necessitates the consideration of the structural connectome’s foundational role in shaping functional connectivity [40]. Although this work is still in its infancy, some interesting findings on sex differences in structural brain graphs based on diffusion MRI have been reported. For example, females displayed stronger features associated with network integration than males (e.g., higher global efficiency) [41], a finding apparently opposite to our observation in the functional connectome. Furthermore, males showed greater diffusion anisotropy and FC in unimodal sensorimotor cortices, whereas females showed greater tract complexity, cortical thickness and FC in the default mode network [13]. It has also been reported that the female structural brain graph was characterized by more edges and spanning trees, a larger minimal bisection width and a better expander graph [42]. However, further investigations that concurrently assess the properties of both functional and structural networks are required to address the important question to what extent FC differences are merely a result of structural differences or may serve as an independent predictor of behavioral differences.

The results presented in this study need to be interpreted in the context of several methodological limitations. (1) The underlying reasons for the observed sex differences, such as, potentially, fluctuations in autonomic system activity [43] or brain structure, remain unknown. They need to be further explored, potentially within a framework of a recently proposed three-layer model [44] that may explain flexible transitions between brain states, slow oscillatory processes and associated dFC parameters, including *MDT* and *Prev*. (2) Sex differences may interact with certain demographic characteristics of the sampled populations. Our two samples consisted of largely healthy young adults. However, the OWN sample also contained participants with mild forms of addictive behavior, which were too few to permit a meaningful investigation of interactions between sex and such behaviors. In addition, dFC parameters have been linked to the pathophysiology of various brain diseases, including depression [45], autism [46] Alzheimer’s disease [47] and multiple sclerosis [48], all of which exhibit notable sex dimorphism. Thus, further studies are needed to investigate potential interactions with age, clinical diagnosis, or other factors, such as cultural environment, gender, or hormone levels. (3) Although we did not observe sex differences in head motion, a prevalent source of time-varying noise, an influence of motion on the results cannot be fully excluded. (4) We used k-means clustering, renowned for its efficiency and robustness, to identify the two brain states. Nonetheless, it is pertinent to recognize its susceptibility to outliers [49]. Explorations into alternative methods for delineating dynamic brain states, e.g., co-activation analyses [50], are warranted to refine techniques for identifying functional connectivity states and state transitions. Methods that consider more than two brain states [3, 16, 51] may be vulnerable to reduced parameter reliability but also more sensitive to specific effects.

In conclusion, we confirmed preregistered hypotheses regarding sex differences in both sFC and dFC measures of brain integration and segregation, with higher values for integration and lower values for segregation in males than in females and consistent effect sizes across the two datasets. dFC parameters offered not only more specificity in this regard (e.g. sex differences in *MDT* but not in *ITI)* but also significant sex differences (in the case of *Prev*_*S*_, *MDT*_*S*_, and *Var*_*I*_) in both samples, unlike the sFC parameters. Taken together, our results underscore the utility and potential of dFC analysis as a valuable tool for probing sex differences in brain function. Future endeavors should extend these inquiries to explore whether these differences in functional brain integration undergo alterations across normal developmental stages or interact with neuropsychiatric disorders, culture, the menstrual cycle, or other factors.

## Supplementary information

Supplementary material 1.

## Data Availability

This study was preregistered after an analysis of the OWN data [22]. All extracted parameters, including time series for connectivity matrices and the required software are openly available at OSF [52]. The raw MRI data from our acquisition (i.e., OWN data) are not available since complex brain images may contain fingerprint-like information, which could lead to reidentification of the subjects. Discussions on how to treat such data at the university level are ongoing. Individual requests for data access can be sent to the corresponding author. OWN data time-courses and matrices are available at OSF [23] including static and dynamic parameters. The code to produce dFC parameters is available on OSF [52]. The HCP data are publicly available [21].
