# Supplementary material for: Sex differences in dynamic and static measures of brain integration derived from resting-state functional magnetic resonance imaging
Source: Biol Sex Differ. 2026 Apr 4;17:76. doi: 10.1186/s13293-026-00891-z (PMC13067390; doi:10.1186/s13293-026-00891-z)
Supplement: Supplementary file 1 — Supplementary material 1. [file 13293_2026_891_MOESM1_ESM.docx]

**Supplementary Information for**

**“Sex Differences in Dynamic and Static Measures of Brain Integration Derived from Resting-state Functional Magnetic Resonance Imaging”**

Xiaojing Fang^1*^, Olivia Schwemmer^2*^, Abigail Hogan^1^, and
Michael Marxen^1^

^1^Department of Psychiatry and Psychotherapy, Technische Universität Dresden, Würzburger Straße 35, 01187 Dresden, Germany

^2^Department of Psychology, Technische Universität Dresden, Würzburger Straße 35, 01187 Dresden, Germany

**Correspondence concerning this article should be addressed to:**

Michael Marxen, Ph.D., Dipl.-Phys.

Section of Systems Neuroscience

Department of Psychiatry and Psychotherapy

Technische Universität Dresden

Würzburger Straße 35

01187 Dresden

Germany

Phone: +49 (351) 46342212

Fax: +49 (351) 46342202

E-Mail: [michael.marxen@tu-dresden.de](mailto:michael.marxen@tu-dresden.de)

**1.1 Parameters of data acquisition**

*OWN data*

Resting-state fMRI data (16 min 27 sec) based on a multi-band oblique-axial (T > C ~ -17°) 2D EPI sequence [1] were acquired with TR 987 ms, TE 32.6 ms, voxel size 2.0 mm × 2.0 mm × 2.0 mm, slice gap 0 mm, FOV 192 mm ×192 mm, multiband factor 6, flip angle 62°, matrix 96 × 96, BW 1860 Hz/Px, 72 interleaved slices, and 1000 volumes.

*HCP data*

For the rs-fMRI data (14 min 24 sec), a simultaneous multi-slice pulse sequence with an acceleration factor of eight [2] was used with TR 720 ms, TE 33.1 ms, voxel size 2.0 mm × 2.0 mm × 2.0 mm, FOV 208 mm × 208 mm, multi-band factor 8, flip angle 52°, matrix 96 × 96, BW 2290 Hz/Px, 72 interleaved slices, and 1200 volumes. We used the data from the two sessions with left-to-right phase-encoding directions.

**1.2 Nuisance regression and filtering**

*OWN data*

After preprocessing, we subsequently regressed out six head motion parameters (i.e., three translations and three rotations), signals of cerebrospinal fluid and white matter. For sFC, we used temporal bandpass filtering with a range of 0.01 ~ 0.1 Hz. For dFC computation, we used filtering with a range of 1/(w*TR) ~ 0.1 Hz, where w = 40, which resulted in a low-frequency boundary of 0.025 Hz [3].

*HCP data*

We used the preprocessed rs-fMRI data with FIX cleaning [4, 5] from the HCP1200 dataset and regressed out the six head motion parameters as well as cerebrospinal fluid and white matter signals. We used the same bandpass filtering parameters as those used for the OWN data for the sFC in the HCP data. For the dFC, the band-pass filtering was employed with the consistent high-frequency boundary of 0.1 Hz and lower-frequency boundary of around 0.025 Hz as used in OWN data, which corresponds to a window width of 55 TR. After data filtering, we calculated the dynamic and static parameters for the two sessions with left-to-right phase-encoding direction in HCP, and averaged the obtained parameters across the two sessions to increase parameter reliability.

**1.3 Atlas for FC analyses**

We used the first version of the Automated Anatomical Labeling atlas (AAL) [6], which consists of 116 regions of interest (ROIs), and grouped it into nine networks on the basis of Yeo’s seven functional networks on the cerebral cortex [7], and anatomical parcellations of the subcortical regions and cerebellum (i.e., visual network, sensory-motor network, dorsal attention network, ventral attention network, limbic network, frontoparietal network, default mode network, basal ganglia network, and cerebellar network). Mean time series were extracted by spatially averaging all voxels within each ROI.

**1.4 Static FC parameters**

The modularity is computed as:

$Q=\sum_{i=1}^{m} (e_{ii}-a_{i}^{2})$ , (1)

where *e_ii_* is the fraction of all edges that connect two nodes within module *i*, *a_i_* is the fraction of edges that connect a node in module *i* to any other node, and *m* is the total number of modules computed via Newman’s modularity algorithm [8]. Global efficiency is defined as:

$E_{global}=\frac{1}{N*\left( N-1 \right)}\sum_{i\neq j} \frac{1}{L_{ij}}$ , (2)

where *N* is the number of nodes, *L_ij_* is the minimum path length between nodes *i* and *j*. In this study, only positive connectivity was used for the calculation.

**1.5 Dynamic FC parameters**

The number of subjects used to compute each parameter is given in Supplementary Table S6. Definition of the dynamic parameters:

1) Mean dwell time (*MDT*) – the average time one participant stayed in state I or S continuously during a run.

2) Prevalence (*Prev*) – a proportion of windows spent in state I or S with respect to the total number of windows within a recording. Note that *Prev_I_ = 1- Prev_S_*; thus, we report primarily results for *Prev_S_* only as effects for *Prev_I_* are merely opposite in sign.

3) Inter-transition interval (*ITI*) – the length of time residing in any state before transitioning to a new state. This measure is very close to the mean of the two MDT times for a two-state model and, thus, not independent. Conceptually, however, it is an interesting measure of how fast a person generally switches states independent of what the states are. It is commonly included in dFC studies with more than two states [9].

4) State variability (*Var*) – the mean Euclidean distance of the state instances belonging to one subject to the run-average of all windows within the same state and subject [10, 11]. This parameter captures the extent to which individual dFC state instances deviate from the within- subject mean, thus, providing a quantitative measure of within-subject heterogeneity or an inverse measure of stability. While it is theoretically possible that this measure could be independent of the other dFC measures, in practice, it is correlated in particular with *Prev* (see Table S5).

In the HCP dataset, where two sessions with left-to-right phase-encoding direction are available, we employed the averaged parameters of these sessions as the final brain state values of the HCP for this study. Note that *MDT, Prev,* and *Var* are specific for the I and S states, whereas ITI is not, resulting in 7 parameters. These parameters provide a comprehensive summary of dFC for a two-state model and are all included here for completeness given that null-findings in large data sets are also valuable scientific observations.


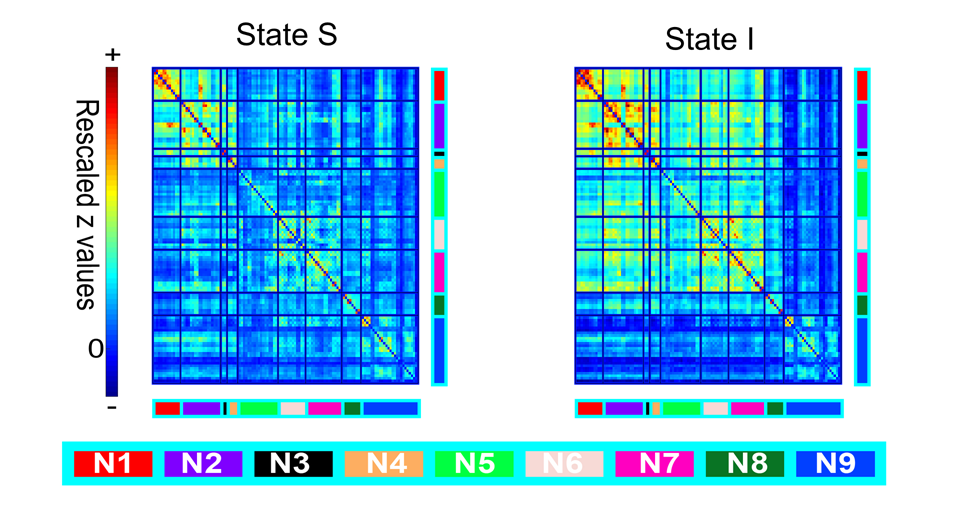


**Figure S1.** Cluster centroids (k = 2) of dFC matrices based on the AAL atlas (Version 1) for OWN data. N1: visual network; N2: sensory-motor network; N3: dorsal attention network; N4: ventral attention network; N5: limbic network; N6: fronto-parietal network; N7: default mode network; N8: basal ganglia network; N9: cerebellum network.


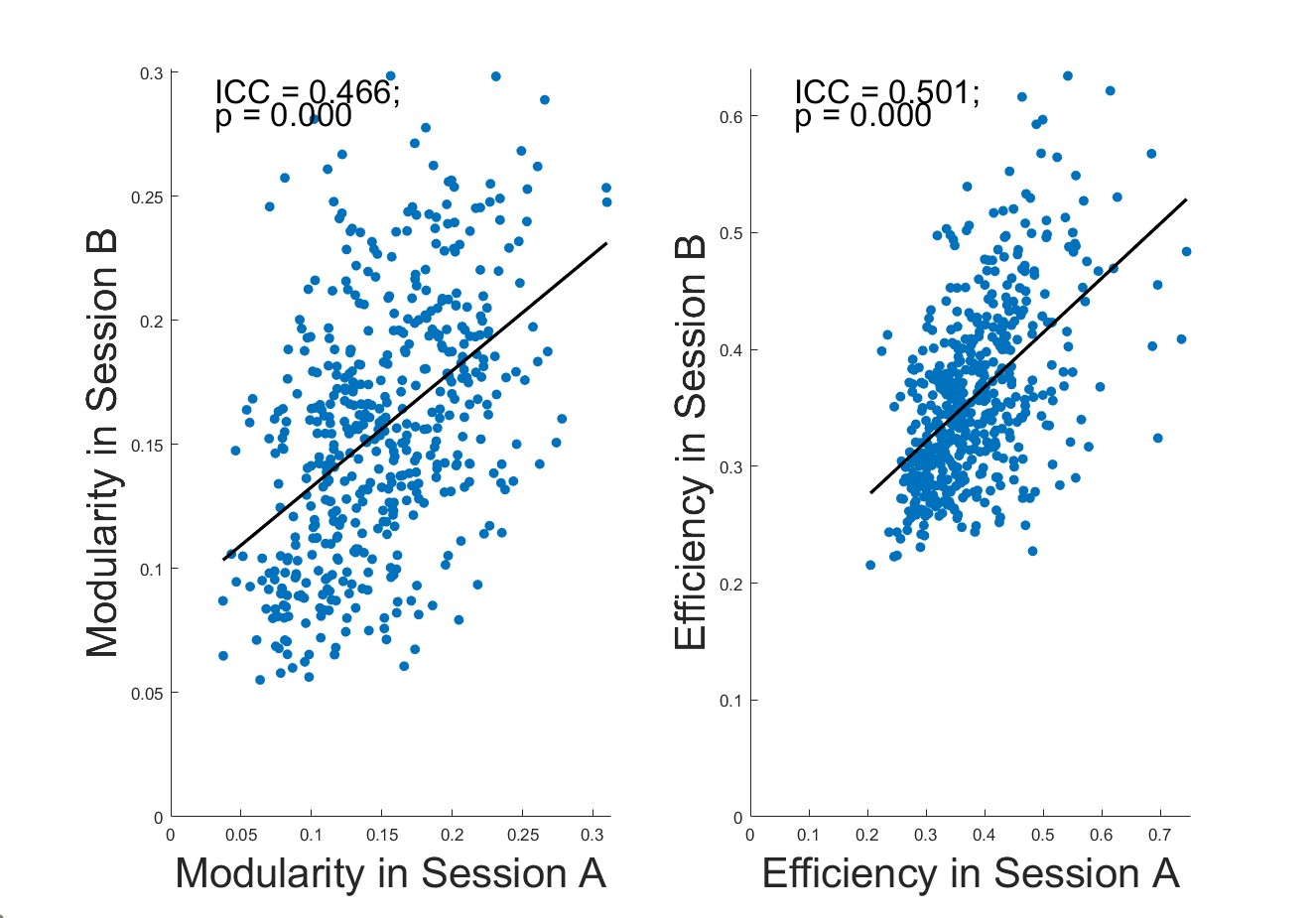


**Figure S2.** Plots of test-retest reliability for the static functional connectivity graph metrics modularity and (global) efficiency. Intra-class correlation coefficients (ICCs) are given.

**Table S1.** Pearson’s correlations *r* between the dynamic and static parameters in OWN and HCP data. *P*-values are uncorrected. *: *p* < 0.05; **: *p* < 0.005.

|  | | ***MDT_I_*** | ***Prev_S_*** | ***ITI*** | ***Var_S_*** | ***Var_I_*** | **Global efficiency** | **Modularity** |
| --- | --- | --- | --- | --- | --- | --- | --- | --- |
| **OWN data** | | | | | | | | |
| ***MDT_S_*** | *r* | -0.343^*^ | 0.576^**^ | 0.337^*^ | 0.268^*^ | -0.351^**^ | -0.382^**^ | 0.214 |
|  | *p* | 0.012 | 0.000 | 0.014 | 0.048 | 0.009 | 0.004 | 0.116 |
|  | N | 53 | 55 | 53 | 55 | 55 | 55 | 55 |
| ***MDT_I_*** | *r* | - | -0.694^**^ | 0.769^**^ | -0.512^**^ | 0.369^**^ | 0.426^**^ | -0.327^*^ |
|  | *p* | - | 0 | 0 | 0 | 0.005 | 0.001 | 0.014 |
|  | N | - | 56 | 53 | 56 | 56 | 56 | 56 |
| ***Prev_S_*** | *r* | - | - | -0.314^*^ | 0.517^**^ | -0.657^**^ | -0.502^**^ | 0.410^**^ |
|  | *p* | - | - | 0.022 | 0 | 0 | 0 | 0.001 |
|  | N | - | - | 53 | 62 | 60 | 63 | 63 |
| ***ITI*** | *r* | - | - | - | -0.356^**^ | 0.164 | 0.167 | -0.189 |
|  | *p* | - | - | - | 0.009 | 0.242 | 0.232 | 0.176 |
|  | N | - | - | - | 53 | 53 | 53 | 53 |
| ***Var_S_*** | *r* | - | - | - | - | -0.193 | -0.339^**^ | 0.259^*^ |
|  | *p* | - | - | - | - | 0.143 | 0.007 | 0.042 |
|  | N | - | - | - | - | 59 | 62 | 62 |
| ***Var_I_*** | *r* | - | - | - | - | - | 0.144 | -0.196 |
|  | *p* | - | - | - | - | - | 0.272 | 0.132 |
|  | N | - | - | - | - | - | 60 | 60 |
| **Global**  **efficiency** | *r* | - | - | - | - | - | - | -0.775^**^ |
|  | *p* | - | - | - | - | - | - | 0 |
|  | N | - | - | - | - | - | - | 63 |
| **HCP data** | | | | | | | | |
| ***MDT_S_*** | *r* | -0.263^**^ | 0.698^**^ | 0.352^**^ | 0.348^**^ | -0.561^**^ | -0.514^**^ | 0.571^**^ |
|  | *p* | 0.000 | 0.000 | 0.000 | 0.000 | 0.000 | 0.000 | 0.000 |
|  | N | 496 | 500 | 496 | 500 | 500 | 500 | 500 |
| ***MDT_I_*** | *r* | - | -0.559^**^ | 0.743^**^ | -0.418^**^ | 0.271^**^ | 0.483^**^ | -0.448^**^ |
|  | *p* | - | 0.000 | 0.000 | 0.000 | 0.000 | 0.000 | 0.000 |
|  | N | - | 497 | 496 | 497 | 497 | 497 | 497 |
| ***Prev_S_*** | *r* | - | - | -0.112^*^ | 0.593^**^ | -0.627^**^ | -0.802^**^ | 0.838^**^ |
|  | *p* | - | - | 0.013 | 0.000 | 0.000 | 0.000 | 0.000 |
|  | N | - | - | 496 | 501 | 501 | 501 | 501 |
| ***ITI*** | *r* | - | - | - | -0.184^**^ | -0.055 | 0.157^**^ | -0.083 |
|  | *p* | - | - | - | 0.000 | 0.223 | 0.000 | 0.065 |
|  | N | - | - | - | 496 | 496 | 496 | 496 |
| ***Var_S_*** | *r* | - | - | - | - | -0.275^**^ | -0.620^**^ | 0.554^**^ |
|  | *p* | - | - | - | - | 0.000 | 0.000 | 0.000 |
|  | N | - | - | - | - | 501 | 501 | 501 |
| ***Var_I_*** | *r* | - | - | - | - | - | 0.517^**^ | -0.616^**^ |
|  | *p* | - | - | - | - | - | 0.000 | 0.000 |
|  | N | - | - | - | - | - | 501 | 501 |
| **Global**  **efficiency** | *r* | - | - | - | - | - | - | -0.793^**^ |
|  | *p* | - | - | - | - | - | - | 0.000 |
|  | N | - | - | - | - | - | - | 501 |

**Table S2.** Results of two-side two-sample t-test between groups for 58-behavioral-task performances in NIH toolbox and bivariate correlation between the performances and *Prev_I_* for HCP. *P*-values are uncorrected. *: *p* < 0.05; **: *p* < 0.005; d*f*: degree of freedom.

| **Parameter** | **t-test** | | **Pearson correlation** | | | | | | | | | | | | |
| --- | --- | --- | --- | --- | --- | --- | --- | --- | --- | --- | --- | --- | --- | --- | --- |
|  | **(males – females)** | | **Sex**  **(male = 1; female = 2)** | | | ***Prev_I_*** | | | **Modularity** | | | **Global efficiency** | | | |
|  | *t-*value (df) | *p* -value | *r* -value | *p-* value | N | *r* -value | *p*-value | N | *r* -value | *p-* value | N | | *r* -value | *p* -value | N |
| InstruSupp_Unadj | -0.995 (498) | 0.320 | 0.045 | 0.320 | 500 | -0.027 | 0.540 | 500 | -0.015 | 0.744 | 500 | | 0.036 | 0.418 | 500 |
| PercStress_Unadj | -1.519 (498) | 0.129 | 0.068 | 0.129 | 500 | 0.024 | 0.597 | 500 | 0.026 | 0.565 | 500 | | -0.028 | 0.530 | 500 |
| SelfEff_Unadj | 2.459 (498) | 0.014* | -0.110 | 0.014* | 500 | 0.050 | 0.267 | 500 | 0.020 | 0.648 | 500 | | -0.012 | 0.789 | 500 |
| PicSeq_Unadj | -1.859 (499) | 0.064 | 0.083 | 0.064 | 501 | -0.041 | 0.355 | 501 | -0.002 | 0.961 | 501 | | 0.031 | 0.487 | 501 |
| CardSort_Unadj | 1.850 (497) | 0.065 | -0.083 | 0.065 | 499 | -0.040 | 0.372 | 499 | -0.044 | 0.332 | 499 | | 0.051 | 0.252 | 499 |
| Flanker_Unadj | 2.779 (499) | 0.006* | -0.123 | 0.006* | 501 | 0.028 | 0.528 | 501 | 0.016 | 0.722 | 501 | | -0.047 | 0.299 | 501 |
| PMAT24_A_CR | 4.864 (494.336) | 0.000** | -0.213 | 0.000** | 497 | 0.102 | 0.023* | 497 | 0.098 | 0.029* | 497 | | -0.089 | 0.047* | 497 |
| ReadEng_Unadj | 3.504 (499) | 0.000** | -0.155 | 0.000** | 501 | -0.004 | 0.936 | 501 | -0.029 | 0.510 | 501 | | 0.006 | 0.893 | 501 |
| PicVocab_Unadj | 2.548 (499) | 0.011* | -0.113 | 0.011* | 501 | -0.028 | 0.537 | 501 | -0.028 | 0.531 | 501 | | 0.029 | 0.512 | 501 |
| ProcSpeed_Unadj | 0.877 (499) | 0.381 | -0.039 | 0.381 | 501 | -0.089 | 0.047* | 501 | -0.069 | 0.121 | 501 | | 0.062 | 0.165 | 501 |
| DDisc_AUC_40K | 1.292 (496) | 0.197 | -0.058 | 0.197 | 498 | -0.038 | 0.402 | 498 | -0.011 | 0.804 | 498 | | 0.008 | 0.852 | 498 |
| VSPLOT_TC | 5.297 (496) | 0.000** | -0.231 | 0.000** | 498 | 0.004 | 0.927 | 498 | 0.039 | 0.381 | 498 | | 0.005 | 0.910 | 498 |
| SCPT_SEN | 0.944 (496) | 0.346 | -0.042 | 0.346 | 498 | 0.009 | 0.834 | 498 | -0.011 | 0.803 | 498 | | -0.002 | 0.973 | 498 |
| SCPT_SPEC | -2.795 (496) | 0.005** | 0.125 | 0.005** | 498 | -0.096 | 0.032* | 498 | -0.060 | 0.178 | 498 | | 0.101 | 0.024* | 498 |
| IWRD_TOT | -1.337 (496) | 0.182 | 0.060 | 0.182 | 498 | -0.043 | 0.337 | 498 | -0.045 | 0.312 | 498 | | 0.054 | 0.229 | 498 |
| ListSort_Unadj | 2.377 (499) | 0.018* | -0.106 | 0.018* | 501 | 0.055 | 0.223 | 501 | 0.011 | 0.806 | 501 | | -0.076 | 0.088 | 501 |
| MMSE_Score | -0.94 (499) | 0.348 | 0.042 | 0.348 | 501 | 0.016 | 0.726 | 501 | 0.061 | 0.176 | 501 | | -0.012 | 0.787 | 501 |
| PSQI_Score | -0.265 (499) | 0.791 | 0.012 | 0.791 | 501 | 0.021 | 0.634 | 501 | -0.011 | 0.804 | 501 | | -0.007 | 0.879 | 501 |
| Endurance_Unadj | 7.124 (437.794) | 0.000** | -0.308 | 0.000** | 501 | 0.010 | 0.824 | 501 | -0.016 | 0.717 | 501 | | 0.042 | 0.350 | 501 |
| GaitSpeed_Comp | -0.787 (499) | 0.431 | 0.035 | 0.431 | 501 | -0.103 | 0.021* | 501 | -0.093 | 0.037* | 501 | | 0.085 | 0.058 | 501 |
| Dexterity_Unadj | -5.157 (499) | 0.000** | 0.225 | 0.000** | 501 | -0.175 | 0.000** | 501 | -0.120 | 0.007* | 501 | | 0.122 | 0.006* | 501 |
| Strength_Unadj | 25.853 (425.138) | 0.000** | -0.762 | 0.000** | 501 | 0.223 | 0.000** | 501 | 0.175 | 0.000** | 501 | | -0.198 | 0.000** | 501 |
| Odor_Unadj | -2.345 (498) | 0.019* | 0.105 | 0.019* | 500 | -0.003 | 0.938 | 500 | 0.030 | 0.497 | 500 | | -0.021 | 0.634 | 500 |
| PainInterf_Tscore | 1.177 (498) | 0.240 | -0.053 | 0.240 | 500 | 0.020 | 0.662 | 500 | 0.035 | 0.436 | 500 | | -0.026 | 0.558 | 500 |
| Taste_Unadj | -4.349 (497) | 0.000** | 0.191 | 0.000** | 499 | -0.020 | 0.659 | 499 | -0.030 | 0.500 | 499 | | -0.003 | 0.940 | 499 |
| Mars_Final | 1.466 (235.638) | 0.144 | -0.069 | 0.122 | 497 | -0.034 | 0.449 | 497 | -0.034 | 0.447 | 497 | | 0.038 | 0.399 | 497 |
| Emotion_Task_Face_Acc | -1.94 (325.943) | 0.053 | 0.090 | 0.045* | 496 | -0.051 | 0.254 | 496 | -0.014 | 0.763 | 496 | | 0.016 | 0.721 | 496 |
| Language_Task_Math_Avg_Difficulty_Level | 1.018 (496) | 0.309 | -0.046 | 0.309 | 498 | 0.086 | 0.054 | 498 | 0.065 | 0.145 | 498 | | -0.071 | 0.111 | 498 |
| Language_Task_Story_Avg_Difficulty_Level | 3.407 (496) | 0.001** | -0.151 | 0.001** | 498 | -0.007 | 0.877 | 498 | 0.024 | 0.598 | 498 | | 0.005 | 0.918 | 498 |
| Relational_Task_Acc | 0.950  (491) | 0.343 | -0.043 | 0.343 | 493 | -0.036 | 0.424 | 493 | 0.006 | 0.898 | 493 | | -0.013 | 0.779 | 493 |
| Social_Task_Perc_Random | -1.533 (496) | 0.126 | 0.069 | 0.126 | 498 | -0.045 | 0.320 | 498 | -0.015 | 0.741 | 498 | | 0.022 | 0.627 | 498 |
| Social_Task_Perc_TOM | 1.704 (496) | 0.089 | -0.076 | 0.089 | 498 | 0.023 | 0.606 | 498 | -0.009 | 0.845 | 498 | | -0.023 | 0.609 | 498 |
| WM_Task_Acc | 1.838 (499) | 0.067 | -0.082 | 0.067 | 501 | -0.070 | 0.120 | 501 | -0.054 | 0.229 | 501 | | 0.076 | 0.088 | 501 |
| NEOFAC_A | -5.676 (497) | 0.000** | 0.247 | 0.000** | 499 | -0.159 | 0.000** | 499 | -0.091 | 0.041* | 499 | | 0.130 | 0.004** | 499 |
| NEOFAC_O | 2.13 (497) | 0.034* | -0.095 | 0.034* | 499 | 0.028 | 0.528 | 499 | 0.018 | 0.693 | 499 | | -0.015 | 0.736 | 499 |
| NEOFAC_C | -2.649 (497) | 0.008* | 0.118 | 0.008* | 499 | -0.090 | 0.045* | 499 | -0.116 | 0.009* | 499 | | 0.088 | 0.050* | 499 |
| NEOFAC_N | -2.622 (467.17) | 0.009* | 0.118 | 0.008* | 499 | 0.005 | 0.906 | 499 | -0.009 | 0.849 | 499 | | 0.001 | 0.990 | 499 |
| NEOFAC_E | -0.822 (497) | 0.411 | 0.037 | 0.411 | 499 | 0.007 | 0.868 | 499 | -0.001 | 0.979 | 499 | | 0.025 | 0.571 | 499 |
| ER40_CR | -1.449 (496) | 0.148 | 0.065 | 0.148 | 498 | -0.030 | 0.499 | 498 | -0.061 | 0.175 | 498 | | 0.044 | 0.331 | 498 |
| ER40ANG | -1.321 (496) | 0.187 | 0.059 | 0.187 | 498 | -0.003 | 0.944 | 498 | -0.060 | 0.178 | 498 | | 0.006 | 0.897 | 498 |
| ER40FEAR | -0.932 (496) | 0.352 | 0.042 | 0.352 | 498 | -0.014 | 0.759 | 498 | 0.003 | 0.951 | 498 | | 0.025 | 0.581 | 498 |
| ER40HAP | -0.849 (496) | 0.397 | 0.038 | 0.397 | 498 | -0.077 | 0.085 | 498 | -0.049 | 0.278 | 498 | | 0.070 | 0.118 | 498 |
| ER40NOE | 1.018 (496) | 0.309 | -0.046 | 0.309 | 498 | 0.012 | 0.791 | 498 | -0.016 | 0.723 | 498 | | -0.015 | 0.745 | 498 |
| ER40SAD | -2.013 (496) | 0.045* | 0.090 | 0.045* | 498 | -0.048 | 0.287 | 498 | -0.058 | 0.196 | 498 | | 0.068 | 0.128 | 498 |
| AngAffect_Unadj | 0.879 (498) | 0.380 | -0.039 | 0.380 | 500 | 0.032 | 0.470 | 500 | 0.034 | 0.443 | 500 | | -0.043 | 0.334 | 500 |
| AngHostil_Unadj | 1.680 (498) | 0.094 | -0.075 | 0.094 | 500 | 0.040 | 0.376 | 500 | 0.052 | 0.250 | 500 | | -0.041 | 0.360 | 500 |
| AngAggr_Unadj | 7.020 (456.44) | 0.000** | -0.303 | 0.000** | 500 | 0.187 | 0.000** | 500 | 0.132 | 0.003** | 500 | | -0.167 | 0.000** | 500 |
| FearAffect_Unadj | -2.323 (498) | 0.021* | 0.104 | 0.021* | 500 | -0.027 | 0.547 | 500 | -0.003 | 0.940 | 500 | | 0.014 | 0.748 | 500 |
| FearSomat_Unadj | -0.328 (498) | 0.743 | 0.015 | 0.743 | 500 | 0.020 | 0.655 | 500 | 0.009 | 0.836 | 500 | | -0.053 | 0.241 | 500 |
| Sadness_Unadj | 0.024 (498) | 0.981 | -0.001 | 0.981 | 500 | 0.023 | 0.601 | 500 | 0.028 | 0.535 | 500 | | -0.007 | 0.881 | 500 |
| LifeSatisf_Unadj | -1.484 (498) | 0.138 | 0.066 | 0.138 | 500 | -0.028 | 0.532 | 500 | -0.002 | 0.973 | 500 | | 0.024 | 0.596 | 500 |
| MeanPurp_Unadj | -2.94 (498) | 0.003** | 0.131 | 0.003** | 500 | -0.036 | 0.427 | 500 | -0.060 | 0.183 | 500 | | 0.047 | 0.296 | 500 |
| PosAffect_Unadj | -0.739 (498) | 0.460 | 0.033 | 0.460 | 500 | -0.033 | 0.463 | 500 | -0.018 | 0.688 | 500 | | 0.026 | 0.569 | 500 |
| Friendship_Unadj | -1.082 (498) | 0.280 | 0.048 | 0.280 | 500 | -0.023 | 0.602 | 500 | -0.037 | 0.404 | 500 | | 0.063 | 0.158 | 500 |
| Loneliness_Unadj | 0.619 (457.246) | 0.536 | -0.028 | 0.532 | 500 | 0.063 | 0.162 | 500 | 0.060 | 0.182 | 500 | | -0.071 | 0.112 | 500 |
| PercHostil_Unadj | 3.250 (498) | 0.001** | -0.144 | 0.001** | 500 | 0.063 | 0.161 | 500 | 0.035 | 0.434 | 500 | | -0.113 | 0.011* | 500 |
| PercReject_Unadj | 1.640 (498) | 0.102 | -0.073 | 0.102 | 500 | 0.026 | 0.567 | 500 | 0.037 | 0.414 | 500 | | -0.08 | 0.073 | 500 |
| EmotSupp_Unadj | -3.100 (498) | 0.002** | 0.138 | 0.002** | 500 | -0.053 | 0.241 | 500 | -0.033 | 0.467 | 500 | | 0.108 | 0.016* | 500 |

**Table S3.** Results of two-side two sample t-test between groups (male vs. female) for alcohol parameters and bivariate correlation between the parameters and *Prev_I_* for HCP. *P*-values are uncorrected. *: *p* < 0.05; **: *p* < 0.005; df: degree of freedom.

| **Parameter** | **t-test** | | **Pearson correlation** | | | | | | | | | | | |
| --- | --- | --- | --- | --- | --- | --- | --- | --- | --- | --- | --- | --- | --- | --- |
|  | **(Males – females)** | | **Sex**  **(male = 1; female = 2)** | | | **Prev_I_** | | | **Modularity** | | | **Global efficiency** | | |
|  | *t-*value (df) | *p* -value | *r* value | *p* -value | N | *r* -value | *p-* value | N | *r* -value | *p-* value | N | *r* -value | *p-* value | N |
| Total_Drinks_7days | 5.694 (320.795) | 0.000 | -0.258** | 0.000 | 494 | 0.067 | 0.134 | 494 | -0.022 | 0.622 | 494 | 0.053 | 0.235 | 494 |
| Num_Days_Drank_7days | 3.58 (457.958) | 0.000 | -0.160** | 0.000 | 499 | 0.038 | 0.402 | 499 | 0.018 | 0.692 | 499 | 0.072 | 0.108 | 499 |
| Avg_Weekday_Drinks_7days | 4.915 (318.945) | 0.000 | -0.225** | 0.000 | 494 | 0.027 | 0.545 | 494 | 0.013 | 0.78 | 494 | 0.027 | 0.556 | 494 |
| Avg_Weekend_Drinks_7days | 4.911 (353.757) | 0.000 | -0.223** | 0.000 | 494 | 0.097* | 0.031 | 494 | -0.059 | 0.192 | 494 | 0.071 | 0.115 | 494 |
| SSAGA_Alc_D4_Dp_Sx | 4.237 (415.300) | 0.000 | -0.190** | 0.000 | 501 | 0.012 | 0.791 | 501 | -0.027 | 0.549 | 501 | 0.027 | 0.542 | 501 |
| SSAGA_Alc_12_Frq | 4.612 (319.204) | 0.000 | -0.213** | 0.000 | 475 | 0.043 | 0.353 | 475 | 0.002 | 0.958 | 475 | 0.018 | 0.698 | 475 |
| SSAGA_Alc_12_Frq_5plus | 6.886 (330.896) | 0.000 | -0.308** | 0.000 | 475 | 0.107* | 0.019 | 475 | − .096* | 0.036 | 475 | 0.096* | 0.036 | 475 |
| SSAGA_Alc_Age_1st_Use | 1.096 (473) | 0.274 | -0.050 | 0.274 | 475 | 0.017 | 0.714 | 475 | -0.004 | 0.934 | 475 | 0.031 | 0.501 | 475 |

**Table S4.** Bootstrapping results (5000 samples) of mediate effect analyses for seven behavioral task performances in NIH toolbox for HCP. Sex: male = 1; female = 2; SD: standard deviation; BootCI: Bootstrapping confidence interval.

| **Effect** | **Estimate (SD)** | **95% BootCI** |
| --- | --- | --- |
| **model 1 (N = 497)** |  |  |
| a: sex–>Prev_I_ | -0.585 (0.086) | [-0.754, -0.414] |
| b: Prev_I_–>PMAT24_A_CR | 0.043 (0.047) | [-0.046, 0.138] |
| c': sex–>PMAT24_A_CR | -0.399 (0.090) | [-0.576, -0.222] |
| a*b: sex–>Prev_I_–>PMAT24_A_CR | -0.025 (0.028) | [-0.084, 0.029] |
| **model 2 (N = 501)** |  |  |
| a: sex–>Prev_I_ | -0.576 (0.085) | [-0.744, -0.408] |
| b: Prev_I_–>Dexterity_Unadj | -0.119 (0.044) | [-0.205, -0.034] |
| c': sex–>Dexterity_Unadj | 0.380 (0.090) | [0.201, 0.556] |
| a*b: sex–>Prev_I_–>Dexterity_Unadj | 0.069 (0.029) | [0.017, 0.129] |
| **model 3 (N = 501)** |  |  |
| a: sex–>Prev_I_ | -0.576 (0.085) | [-0.744, -0.408] |
| b: Prev_I_–>Strength_Unadj | 0.004 (0.028) | [-0.053, 0.058] |
| c': sex–>Strength_Unadj | -1.521 (0.059) | [-1.636, -1.400] |
| a*b: sex–>Prev_I_–>Strength_Unadj | -0.002 (0.016) | [-0.034, 0.030] |
| **model 4 (N = 499)** |  |  |
| a: sex–>Prev_I_ | -0.584 (0.086) | [-0.753, -0.419] |
| b: Prev_I_–>NEOFAC_A | -0.094 (0.046) | [-0.185, -0.004] |
| c': sex–>NEOFAC_A | 0.438 (0.092) | [0.256, 0.619] |
| a*b: sex–>Prev_I_–>NEOFAC_A | 0.055 (0.028) | [0.002, 0.114] |
| **model 5 (N = 499)** |  |  |
| a: sex–>Prev_I_ | -0.584 (0.086) | [-0.753, -0.419] |
| b: Prev_I_–>NEOFAC_C | -0.060 (0.048) | [-0.153, 0.036] |
| c': sex–>NEOFAC_C | 0.201 (0.096) | [0.015, 0.389] |
| a*b: sex–>Prev_I_–>NEOFAC_C | 0.035 (0.029) | [-0.021, 0.094] |
| **model 6 (N = 500)** |  |  |
| a: sex–>Prev_I_ | -0.582 (0.085) | [-0.749, -0.413] |
| b: Prev_I_–>AngAggr_Unadj | 0.109 (0.045) | [0.02, 0.198] |
| c': sex–>AngAggr_Unadj | -0.542 (0.088) | [-0.717, -0.375] |
| a*b: sex–>Prev_I_–>AngAggr_Unadj | -0.063 (0.028) | [-0.121, -0.011] |

**Table S5.** Correlations of *Var_S_* with *Prev_S_* and *MDT_S_* after controlling for sex. *: *p* < 0.05; df: degree of freedom; *p*-values are uncorrected.

|  | | ***MDT_S_*** | ***MDT_I_*** | ***Prev_S_*** |
| --- | --- | --- | --- | --- |
| **OWN data** | | | | |
| ***Var_S_*** | *rho* -value | 0.199 | -0.503 | 0.543 |
|  | *p*-value | 0.157 | 0.000 | 0.000 |
|  | df | 50 | 50 | 50 |
| ***Var_I_*** | *rho-*value | -0.296 | 0.379 | -0.662 |
|  | *p-*value | 0.033 | 0.006 | 0.000 |
|  | df | 50 | 50 | 50 |
| **HCP data** | | | | |
| ***Var_S_*** | *rho*-value | 0.349 | -0.404 | 0.605 |
|  | *p*-value | 0.000 | 0.000 | 0.000 |
|  | df | 493 | 493 | 493 |
| ***Var_I_*** | *rho*-value | -0.537 | 0.269 | -0.635 |
|  | *p*-value | 0.000 | 0.000 | 0.000 |
|  | df | 493 | 493 | 493 |

**Table S6.** Numbers of subjects that did not allow the computation of a particular dynamic FC parameter due to insufficient switches between states (i.e., NaN) and outliers. Outliers were defined as values outside the mean ± 3 standard deviations and included in the analyses.

|  | NaN | outliers |
| --- | --- | --- |
| OWN |  |  |
| *MDT_S_* | 8 | 2 |
| MDT_I_ | 7 | 2 |
| Prev_S_ | 0 | 0 |
| ITI | 10 | 2 |
| Var_S_ | 1 | 0 |
| Var_I_ | 3 | 0 |
| HCP |  |  |
| MDT_S_ | 1 | 8 |
| MDT_I_ | 4 | 9 |
| Prev_S_ | 0 | 0 |
| ITI | 5 | 10 |
| Var_S_ | 0 | 0 |
| Var_I_ | 0 | 0 |

**Reference**

1. Moeller S, Yacoub E, Olman CA, Auerbach E, Strupp J, Harel N, et al. Multiband multislice GE-EPI at 7 tesla, with 16-fold acceleration using partial parallel imaging with application to high spatial and temporal whole-brain fMRI. Magn Reson Med. 2010;63:5:1144-53; doi:10.1002/mrm.22361.

2. Ugurbil K, Xu J, Auerbach EJ, Moeller S, Vu AT, Duarte-Carvajalino JM, et al. Pushing spatial and temporal resolution for functional and diffusion MRI in the Human Connectome Project. Neuroimage. 2013;80:80–104; doi:10.1016/j.neuroimage.2013.05.012.

3. Leonardi N, Van De Ville D. On spurious and real fluctuations of dynamic functional connectivity during rest. Neuroimage. 2015;104:430-6; doi:10.1016/j.neuroimage.2014.09.007.

4. Glasser MF, Sotiropoulos SN, Wilson JA, Coalson TS, Fischl B, Andersson JL, et al. The minimal preprocessing pipelines for the Human Connectome Project. Neuroimage. 2013;80:105 − 24; doi:10.1016/j.neuroimage.2013.04.127.

5. Barch DM, Burgess GC, Harms MP, Petersen SE, Schlaggar BL, Corbetta M, et al. Function in the human connectome: task-fMRI and individual differences in behavior. Neuroimage. 2013;80:169 − 89; doi:10.1016/j.neuroimage.2013.05.033.

6. Tzourio-Mazoyer N, Landeau B, Papathanassiou D, Crivello F, Etard O, Delcroix N, et al. Automated anatomical labeling of activations in SPM using a macroscopic anatomical parcellation of the MNI MRI single-subject brain. Neuroimage. 2002;15:1:273 − 89; doi:10.1006/nimg.2001.0978.

7. Yeo BT, Krienen FM, Sepulcre J, Sabuncu MR, Lashkari D, Hollinshead M, et al. The organization of the human cerebral cortex estimated by intrinsic functional connectivity. J Neurophysiol. 2011;106:3:1125-65; doi:10.1152/jn.00338.2011.

8. Newman ME, Girvan M. Finding and evaluating community structure in networks. Phys Rev E Stat Nonlin Soft Matter Phys. 2004;69:2 Pt 2:026113; doi:10.1103/PhysRevE.69.026113.

9. de Lacy N, McCauley E, Kutz JN, Calhoun VD. Sex-related differences in intrinsic brain dynamism and their neurocognitive correlates. Neuroimage. 2019;202:116116; doi:10.1016/j.neuroimage.2019.116116.

10. Fang X, Marxen M. Test-retest reliability of dynamic functional connectivity parameters for a two-state model. Network Neuroscience. 2025;9:1:371 − 91; doi:10.1162/netn_a_00437.

11. Fang X, Schwemmer O, Bottino M, Marxen M. Sex Differences in Dynamic and Static Measures of Brain Integration derived from Resting State fMRI. Open Science Framework. 2024. doi:https://osf.io/p8usv.
